# MOF-Derived ZrO_2_-Supported Bimetallic Pd–Ni Catalyst for Selective Hydrogenation of 1,3-Butadiene

**DOI:** 10.3390/molecules29102217

**Published:** 2024-05-09

**Authors:** Ying Liu, Lili Liu, Leyuan Wang, Miaoliang Zang, Lei Li, Yunkai Zhang

**Affiliations:** School of Chemistry & Chemical Engineering and Environmental Engineering, Weifang University, Weifang 261061, China; liuying@wfu.edu.cn (Y.L.); 15264543037@163.com (L.W.); 13356710254@163.com (M.Z.); 15863610289@163.com (L.L.); 17753624747@163.com (Y.Z.)

**Keywords:** MOF-derived, ZrO_2_, bimetallic catalysts, Pd–Ni catalysts, hydrogenation of 1,3-butadiene

## Abstract

A series of MOF-derived ZrO_2_-supported Pd-Ni bimetallic catalysts (PdNi/UiO-67-CTAB(n)-A500) were prepared by co-impregnation and pyrolysis at 500 °C under air atmosphere using UiO-67-CTAB(n) (CTAB: cetyltrimethylammonium bromide; n: the concentration of CTAB; *n* = 0, 3, 8, 13, 18) as a sacrificial template. The catalytic activity of PdNi/UiO-66-CTAB(n)-A500 in 1,3-butadiene hydrogenation was found to be dependent on the crystal morphology of the UiO-67 template. The highest activity was observed over the PdNi/UiO-67-CTAB(3)-A500 catalyst which was synthesized using UiO-67-CTAB(3) with uniform octahedral morphology as the template for the 1,3-butadiene selective hydrogenation. The 1,3-butadiene conversion and total butene selectivity were 98.4% and 44.8% at 40 °C within 1 h for the PdNi/UiO-67-CTAB(3)-A500 catalyst, respectively. The catalyst of PdNi/UiO-67-CTAB(3)-A500 can be regenerated in flowing N_2_ at 200 °C. Carbon deposited on the surface of the catalyst was the main reason for its deactivation. This work is valuable for the high-efficiency bimetallic catalyst’s development on the selective hydrogenation of 1,3-butadiene.

## 1. Introduction

C_4_ olefin is an important raw material for the synthesis of polymers [1,2]. However, the C_4_ olefin stream from petroleum cracking contains a trace amount of 1,3-butadiene, which can severely reduce the quality of polymers and easily poison the catalysts for polymerization [2,3,4]. The selective hydrogenation of 1,3-butadiene is an important industrial process to remove 1,3-butadiene from petroleum cracking [5,6,7]. Currently, Pd catalysts are mostly used as heterogeneous catalysts for 1,3-butadiene selective hydrogenation due to their high hydrogenation activity [8,9,10,11]. However, the scarcity and high price of Pd greatly limits its application [12]. Moreover, Pd catalysts generally have low selectivity to total butenes (1-butene, trans-2-butene, and cis-2-butene) at high 1,3-butadiene conversion due to their high hydrogen activation [6,13]. To decrease the cost of the Pd catalyst and gain the high selectivity of butenes, alloying Pd with a second metal (such as Ni, Cu, Co, etc.) and selecting suitable supports to form bimetallic supported catalysts have received considerable attention [2,4,14,15,16,17]. The addition of the second metal in bimetallic Pd-based catalysts can change the surface structure and electronic properties of Pd catalysts, decrease the adsorption capacity of butenes, and improve the selectivity to total butenes [18,19]. Bimetallic Pd-Ni [13,20,21], Pd-Cu [2], Pd-Cr [22], Pd-Au [23], and Pd-Ag [24] catalysts have been shown to have superior butene selectivity over monometallic Pd catalysts. Huang et al. [13] synthesized the Ni_1_-Pd_1_/ZnO catalyst by the bioreduction route using Cinnamomum Camphora leaf extract. They found that the butene selectivity (90.0%) of the bimetallic Ni_1_-Pd_1_/ZnO catalyst was better than that of the monometallic Pd/ZnO catalyst (46.9%) [13]. Yang et al. [2] also reported that the γ-Al_2_O_3_-supported Pd–Cu bimetallic catalyst displayed higher butene selectivity as compared with the monometallic Pd catalyst at the same 1,3-butadiene conversion. Huang et al. [24] reported that the Pd-Ag/ES (ES = eggshell) catalyst displayed high butene selectivity (95.8%) at high 1,3-butadiene conversion (95%) at 45 °C. The bimetallic catalyst Au(2)Pd(1)/MIL-101(Cr) (95.7%) displayed a higher selectivity of total butenes than that of Pd/MIL-101(Cr) (35.0%) when the 1,3-butadiene conversion was about 100% [23].

In recent years, metal–organic frameworks (MOFs) have emerged as a class of promising precursors or sacrificial templates for synthesizing various porous metal oxides or carbon with complex compositions and a unique structure via pyrolysis owing to abundant intrinsic molecular metal sites, an ordered pore structure, high porosity, and an adjustable pore size [25,26,27,28,29,30]. The composition and structure of MOF derivatives can be easily realized by adjusting the precursors or sacrificial templates of MOFs, modifying the pyrolysis temperature, and changing the pyrolysis atmosphere (inert or air atmosphere) [26,29,31,32]. Moreover, the ordered pore structure of MOFs can envelop other metal salts or organic species which could further modify MOF derivatives [26,33]. Recently, MOF derivatives have been widely used in heterogeneous catalysis, especially those using MOF derivatives as a support for metal nanoparticles [34,35,36,37,38]. Zeng et al. [34] synthesized the hierarchical mesoporous CuO@NiO (M-CuO@NiO) catalyst using MOFs as the precursor. The M-CuO@NiO catalyst displayed excellent catalytic activity and selectivity for the deoxygenation of fatty acids [34]. The conversion of stearic acid and the selectivity of C_8_-C_18_ alkanes reached 99.9% and 94.4%, respectively, which were comparable to the Pt/C catalyst [34]. Lei et al. [39] successfully synthesized M-Co_1_Y_1_O_x_ (Y = Cu, Mn, Fe, and Ni) catalysts using Y-doped ZSA-1 as a precursor via pyrolysis at 350 °C. The dopants displayed a great effect on the catalytic performance for toluene catalytic destruction on Co_3_O_4_ derived from Co-MOF [39]. They found that M-Co_1_Cu_1_O_x_ showed the most prominent activity with T_90%_ reduced 31 °C (compared to M-Co_3_O_4_) due to improved physical properties [39]. They also reported that Co-Mn metal oxides synthesized through the pyrolysis of Mn-doped ZSA-1 presented excellent catalytic performance for toluene catalytic oxidation [40]. Wang et al. [41] prepared the nitrogen-doped carbon material (NC-E) from ZIF-8 using a novel and simple method assisted by eutectic salts. They also prepared carbon materials NC-T from ZIF-8 using traditional calcination [41]. The NC-E-supported Pt-Sn catalyst exhibited improved electrochemical activity and stability for the ethanol oxidation reaction than NC-T- and carbon black-supported Pt-Sn catalysts [41]. Wang et al. [42] found that MOF-derived CeO_2_-supported Ag catalysts (Ag-Ce-BTC-C) prepared via one-pot and pyrolysis at 500 °C under Ar and O_2_ atmosphere displayed good catalytic performance for the toluene oxidation reaction (T_90_ = 226 °C).

Among the various types of MOFs, Zr-based MOFs have been extensively studied by catalysis scholars owing to the strong Zr-O coordination bond, feasible synthesis conditions, and the ease of reproducibility [43,44]. UiO-67 is one of the Zr-MOFs, which consists of Zr_6_(OH)_4_O_4_ clusters and 4,4-biphenyl dicarboxylic acid (H_2_bpdc) linkers [45,46]. In the present study, a series of UiO-67-CTAB(n) (CTAB: cetyltrimethylammonium bromide; n: the concentration of CTAB; *n* = 0, 3, 8, 13, 18) with different physicochemical properties (crystal morphology, crystal size, BET specific surface area, etc.) were prepared by the surfactant CTAB assistant solvothermal method. PdNi/UiO-67-CTAB(n)-A500 catalysts were obtained by co-impregnation and pyrolysis at 500 °C under air atmosphere using UiO-67-CTAB(n) as a sacrificial template. The effect of various factors such as the physicochemical property of the MOF template, reaction temperature, and reaction time on the hydrogenation of 1,3-butadiene was studied. A 1,3-butadiene hydrogenation test found that PdNi/UiO-67-CTAB(3)-A500 presented the best catalytic performance for 1,3-butadiene hydrogenation. The recoverability and reusability of the PdNi/UiO-67-CTAB(3)-A500 catalyst were also investigated.

## 2. Results and Discussion

### 2.1. Synthesis and Characterization

**Scheme 1:**
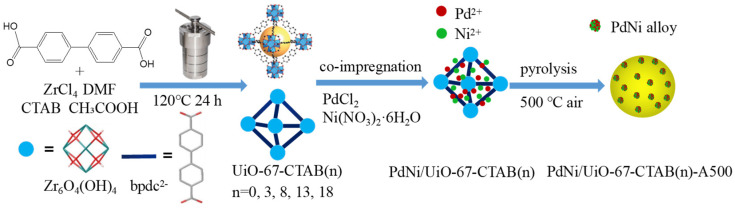
Schematic illustration of PdNi/UiO-67-CTAB(n)-A500 (*n* = 0, 3, 8, 13, 18) catalysts.

The synthesis procedure was illustrated in a schematic representation (Scheme 1). UiO-67-CTAB(n) (*n* = 0, 3, 8, 13, 18) was simply synthesized from ZrCl_4_, H_2_bpdc, acetic acid, CTAB, and DMF by the surfactant CTAB assistant solvothermal method using different CTAB concentrations. Then, the PdNi/UiO-67-CTAB(n)-A500 (*n* = 0, 3, 8, 13, 18) catalyst was synthesized by the co-impregnation and pyrolysis method using PdCl_2_ and Ni(NO_3_)_2_·6H_2_O as the metal precursor.

**Figure 1:**
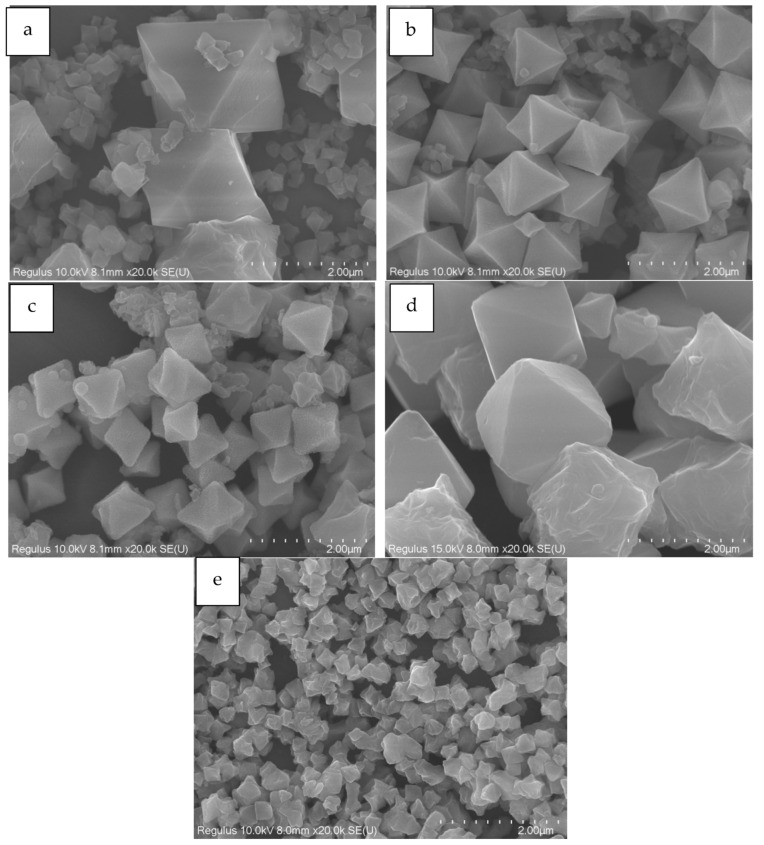
SEM photographs of UiO-67-CTAB(0) (**a**), UiO-67-CTAB(3) (**b**), UiO-67-CTAB(8) (**c**), UiO-67-CTAB(13) (**d**), and UiO-67-CTAB(18) (**e**).

Viewing SEM images, the crystal morphology of UiO-67-CTAB(0) contains both regular octahedral crystals and irregular polyhedral crystals, and it shows a broad crystal size, which ranged from 200 to 2800 nm as a result of the high-degree intergrowth and aggregation of primary crystals (Figure 1) [47]. After adding CTAB to the synthesis solution, UiO-67-CTAB(3) exhibited a uniform octahedral morphology with a crystal size of ca. 1500 nm, which was consistent with the morphology of UiO-67 reported in the literature [48,49]. During the process of crystallization, CTAB was adsorbed on the surface of the UiO-67 primary crystal, which would decrease the van der Waals force and surface energy, promoting the ordered agglomeration, thus forming a more regular morphology [50,51,52]. The long hydrocarbon chains of CTAB also repelled each other, preventing the further interaction with other UiO-67 crystals and excessive growth of UiO-67, resulting in a smaller particle size [53,54]. However, further increasing the concentration of CTAB in the synthetic solution, irregular polyhedral crystals appeared in the synthesized crystals. The percent of irregular polyhedral crystals increased with the increase in CTAB concentration, up to about 100% when the CTAB concentration was 18 g/L. The crystal particle sizes of UiO-67-CTAB(8), UiO-67-CTAB(13), and UiO-67-CTAB(18) were ca. 1100 nm, 2300 nm, and 300 nm, respectively. The concentration of CTAB in synthetic mixture also had a significant effect on the particle size, and the crystal size was varied as follows: UiO-67-CTAB(13) > UiO-67-CTAB(3) > UiO-67-CTAB(8) >UiO-67-CTAB(18) > UiO-67-CTAB(0). These results indicated that excessive CTAB has a negative effect on the formation of a uniform morphology. This may be owing to the fact that most CTAB existed as micelles, rather than dispersed forms, which did not interact well with surfaces [51,55]. Wang et al. [52] also reported that the morphology and size of 146S@ZIF-8 can be changed by adding CTAB. Jubeer et al. [56] reported that the particle size of ZnS could be adjusted by changing the concentration of CTAB. Ping et al. [57] found that NiPO was of a flake shape at a low CTAB:P molar ratio, and the samples began to appear with a tubular structure with the increase in the CTAB:P molar ratio.

**Figure 2:**
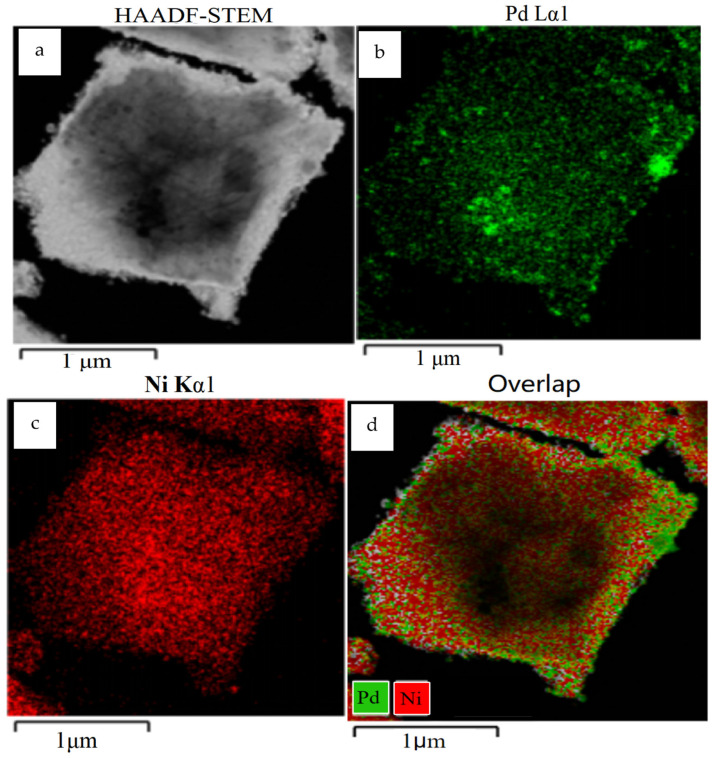
HAADF-STEM (**a**) and EDS mappings (**b**–**d**) of PdNi/UiO-67-CTAB(3)-A500.

The TEM images and Pd–Ni nanoparticle size distribution histograms of PdNi/UiO-67-CTAB(n)-A500 (*n* = 0, 3, 8, 13, 18) catalysts are displayed in Figure S1. As shown in Figure S1, the PdNi/UiO-67-CTAB(0)-A500, PdNi/UiO-67-CTAB(3)-A500, and PdNi/UiO-67-CTAB(18)-A500 catalysts had a narrow nanoparticle size distribution with an average particle size of 6.0, 6.7, and 6.0 nm, respectively. However, PdNi/UiO-67-CTAB(8)-A500 and PdNi/UiO-67-CTAB(13)-A500 showed broader nanoparticle size distribution with an average particle size of 15.3 and 9.2 nm. The characterization of the catalysts by TEM displayed that PdNi/UiO-67-CTAB(8)-A500 presented the largest average particle size, PdNi/UiO-67-CTAB(13)-A500 presented the middle average particle size, and the PdNi/UiO-67-CTAB(0)-A500, PdNi/UiO-67-CTAB(3)-A500, and PdNi/UiO-67-CTAB(18)-A500 catalysts presented smaller average particles sizes. The HAADF-STEM and EDS mapping of the PdNi/UiO-67-CTAB(3)-A500 catalyst are displayed in Figure 2. The HAADF-STEM and EDS mapping of PdNi/UiO-67-CTAB(3)-A500 demonstrated that Pd and Ni elements were highly intermixed and evenly distributed on the support, confirming the formation of Pd-Ni alloys [58,59].

**Figure 3:**
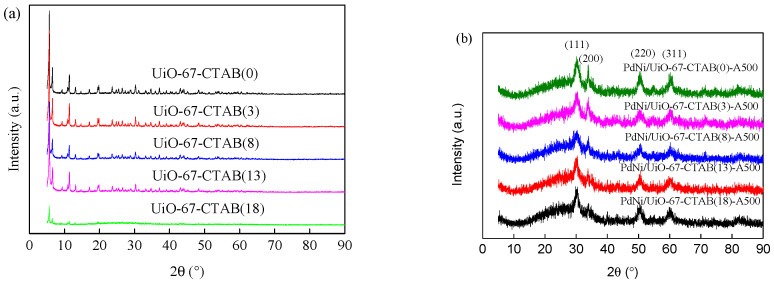
PXRD patterns of UiO-67-CTAB(n) (**a**) and PdNi/UiO-67-CTAB(n)-A500 (**b**) (*n* = 0, 3, 8, 13, 18).

The samples of UiO-67-CTAB(n) and PdNi/UiO-67-CTAB(n)-A500 (*n* = 0, 3, 8, 13, 18) have been characterized by PXRD. The PXRD patterns of UiO-67-CTAB(n) and PdNi/UiO-67-CTAB(n)-A500 (*n* = 0, 3, 8, 13, 18) are presented in Figure 3. PXRD analysis displayed that all the 2θ peaks of UiO-67-CTAB(0) corresponded well with UiO-67 reported in the literature, illustrating that UiO-67-CTAB(0) has been successfully synthesized [44,60,61]. The PXRD peaks of the synthesized UiO-67-CTAB(3), UiO-67-CTAB(8), UiO-67-CTAB(13), and UiO-67-CTAB(18) were almost identical to UiO-67-CTAB(0), which indicates that the synthesized samples had the original UiO-67-CTAB(0) structure (Figure 3a). However, the PXRD peak intensity of UiO-67-CTAB(18) decreased significantly, revealing that its crystallinity was significantly lost (Figure 3a). After pyrolysis at 500 °C under air, the PXRD patterns changed completely (Figure 3b). There was no characteristic diffraction peak from UiO-67 for the PdNi/UiO-67-CTAB(n)-A500 (*n* = 0, 3, 8, 13, 18) catalysts, indicating that UiO-67 was completely decomposed [62]. The PdNi/UiO-67-CTAB(n)-A500 (*n* = 0, 3, 8, 13, 18) catalysts all exhibited four diffraction peaks at 2θ values of 30.15, 33.84, 50.57, and 60.30° which could be assigned to the (111), (200), (220), and (311) crystallographic planes of tetragonal ZrO_2_ (t-ZrO_2_), respectively [44,62,63,64,65,66]. However, the characteristic PXRD diffraction peaks of the PdNi alloy were not detected in PdNi/UiO-67-CTAB(n)-A500 (*n* = 0, 3, 8, 13, 18) due to the low amounts of Pd and Ni and the high dispersion of PdNi alloy nanoparticles on the UiO-67-CTAB(n) (*n* = 0, 3, 8, 13, 18) supports [67,68].

**Figure 4:**
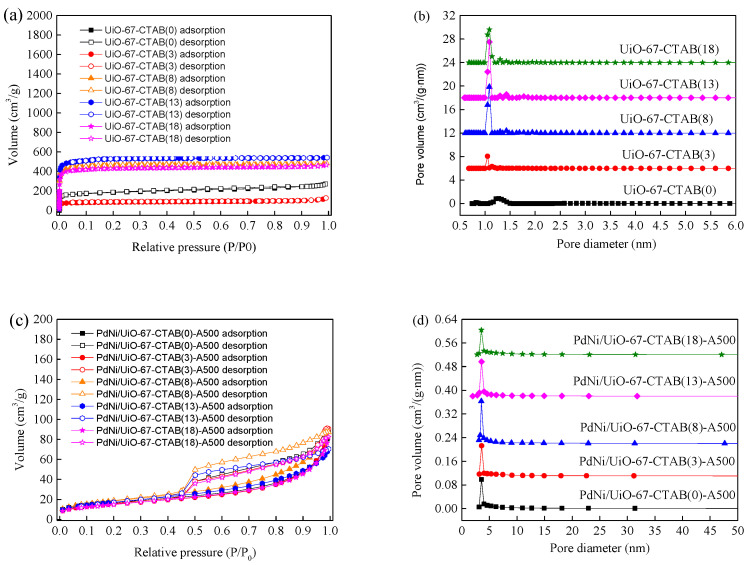
(**a**) N_2_-sorption isotherms of UiO-67-CTAB(n) (*n* = 0, 3, 8, 13, 18); (**b**) pore diameter distributions of UiO-67-CTAB(n) (*n* = 0, 3, 8, 13, 18) calculated using DFT method; (**c**) N_2_-sorption isotherms of PdNi/UiO-67-CTAB(n)-A500 (*n* = 0, 3, 8, 13, 18); (**d**) pore diameter distributions of PdNi/UiO-67-CTAB(n)-A500 (*n* = 0, 3, 8, 13, 18) calculated using DFT method.

UiO-67-CTAB(n) and PdNi/UiO-67-CTAB(n)-A500 (*n* = 0, 3, 8, 13, 18) have been characterized by nitrogen adsorption–desorption at 77 K to estimate the specific surface area, porosity volume, and pore diameter. The specific areas and pore size distributions were determined using the Brunauer–Emmett–Teller (BET) method and density functional theory (DFT) method, respectively [69]. The nitrogen sorption isotherms and pore size distribution diagrams are displayed in Figure 4, and the BET surface area and pore volume dates are displayed in Table 1. As shown in Figure 4a, UiO-67-CTAB(n) (*n* = 0, 3, 8, 13, 18) displayed a pronounced type-I isotherm, indicating the existence of micropores [44]. The DFT-computed pore size distributions displayed maximum values at 0.82 and 1.27 nm for UiO-67-CTAB(0) (Figure 4b). The pore diameters determined by the DFT method were 1.05, 1.09, 1.09, and 1.09 nm for UiO-67-CTAB(3), UiO-67-CTAB(8), UiO-67-CTAB(13), and UiO-67-CTAB(18), respectively (Figure 4b). However, PdNi/UiO-67-CTAB(n)-A500 (*n* = 0, 3, 8, 13, 18) displayed a type-IV isotherm, indicating the emergence of a mesoporous structure after the loading of Pd-Ni nanoparticles and pyrolysis at 500 °C (Figure 4c) [70,71]. The pore diameters based on the BJH model were 3.57, 3.59, 3.53, 3.58, and 3.54 nm for PdNi/UiO-67-CTAB(0)-A500, PdNi/UiO-67-CTAB(3)-A500, PdNi/UiO-67-CTAB(8)-A500, PdNi/UiO-67-CTAB(13)-A500, and PdNi/UiO-67-CTAB(18)-A500, respectively (Figure 4d). The BET surface areas were 686.1, 264.9, 1402.2, 1611.8, and 1309.1 m^2^/g for UiO-67-CTAB(0), UiO-67-CTAB(3), UiO-67-CTAB(8), UiO-67-CTAB(13), and UiO-67-CTAB(18), respectively (Table 1). The total pore volumes were 0.4, 0.17, 0.73, 0.83, and 0.70 cm^3^/g for UiO-67-CTAB(0), UiO-67-CTAB(3), UiO-67-CTAB(8), UiO-67-CTAB(13), and UiO-67-CTAB(18), respectively (Table 1). The BET surface areas and pore volumes were varied as follows: UiO-67-CTAB(13) > UiO-67-CTAB(8) > UiO-67-CTAB(18) > UiO-67-CTAB(0) > UiO-67-CTAB(3). As shown in SEM, the crystal size showed the order UiO-67-CTAB(13) > UiO-67-CTAB(3) > UiO-67-CTAB(8) > UiO-67-CTAB(18) > UiO-67-CTAB(0) (Figure 1). UiO-67-CTAB(3) exhibited a uniform octahedral morphology, and the percent of irregular polyhedral crystals increased with the increase in CTAB concentration. The above results indicated that the BET surface area and pore volume were not only affected by the regularity of the morphology but also by the crystal size. Osuga et al. [72] reported that the BET surface area and pore volume were greatly affected by the morphology of aluminosilicate zeolites. The formation of sheet-like particles increased the BET surface area and decreased the micropore volume [72]. Li et al. [73] found that the different BET surface area of 2D metal–organic frameworks CuBDC (BDC: 1,4-benzenedicarboxylic) and CuBDC-NBA (NBA:4-n-butylbenzoic acid) were induced by their different morphology and size. The BET surface areas and pore volumes of PdNi/UiO-67-CTAB(n)-A500 (*n* = 0, 3, 8, 13, 18) catalysts after Pd-Ni nanoparticle loading and pyrolysis at 500 °C decreased significantly compared to UiO-67-CTAB(n) (*n* = 0, 3, 8, 13, 18). This may be due to the fact that UiO-67 completely decomposed and formed t-ZrO_2_. The PdNi/UiO-67-CTAB(n)-A500 (*n* = 0, 3, 8, 13, 18) catalysts displayed a similar BET surface area (54.3–66.7 m^2^/g) and pore volumes (0.09–0.11 cm^3^/g), illustrating that the properties (BET surface area, pore diameter, and pore volume) of the MOF template have little influence on the BET surface area and pore volume of the catalysts obtained by pyrolysis.

**Figure 5:**
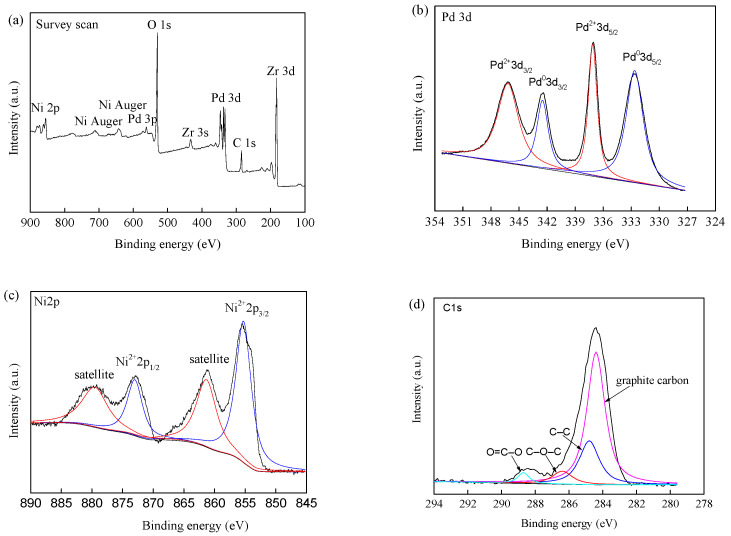
XPS spectra of survey scan (**a**), Pd 3d (**b**), Ni 2p (**c**), and C1s (**d**) for PdNi/UiO-67-CTAB(3)-A500.

**Figure 6:**
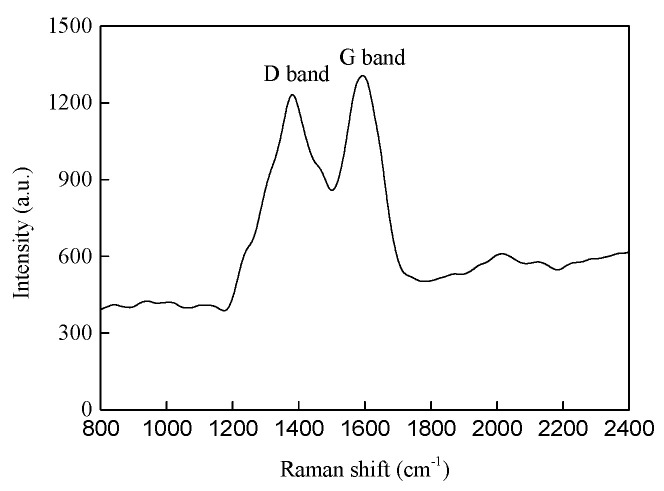
Raman spectra of PdNi/UiO-67-CTAB(3)-A500.

The elemental composition and chemical valence state of the PdNi/UiO-67-CTAB(3)-A500 catalyst were studied by XPS. Figure 5 presents the full XPS spectra along with the Pd 3d, Ni 3p, and C1s narrow scan spectra of PdNi/UiO-67-CTAB(3)-A500. It can be seen in the full survey scan spectra that the PdNi/UiO-67-CTAB(3)-A500 catalyst was mainly composed of Pd, Ni, Zr, O, and C (Figure 5a). The Pd 3d spectrum of PdNi/UiO-67-CTAB(3)-A500 can be deconvoluted into four peaks; the binding energies at 332.7 and 342.5 eV can be ascribed to Pd^0^ 3d_5/2_ and Pd^0^ 3d_3/2_, whereas the two peaks at 337.1 and 346.2 eV can be ascribed to Pd^2+^ 3d_5/2_ and Pd^2+^ 3d_3/2_ (Figure 5b) [20,74]. For the Ni 3p spectrum of PdNi/UiO-67-CTAB(3)-A500, a peak at 855.3 eV as well as a satellite peak at 861.4 eV were apparent in the Ni^2+^ 2p_3/2_ region, whereas a signal at 873.1 eV as well as a satellite peak at 879.6 eV were observed in the Ni^2+^ 2p_5/2_ region (Figure 5c) [34,75]. As shown in C1s peaks, the strong peak at 284.4 eV was attributed to the graphite carbon, and the peaks at 284.8 eV, 286.4 eV, and 288.7 eV corresponded to the calibration peak C–C, C–O–C, and O = C–O, respectively (Figure 5d) [76,77]. The Raman spectra of PdNi/UiO-67-CTAB(3)-A500 displayed two obvious peaks at 1381 cm^−1^ (D band) and 1592 cm^−1^ (G band) which can be attributed to the amorphous carbon species and highly symmetrical and ordered graphitized carbon species (Figure 6) [78,79]. Both the results of XPS and Raman spectroscopy for PdNi/UiO-67-CTAB(3)-A500 illustrated the existence of carbon species; this may be due to the incomplete combustion of catalysts.

### 2.2. Catalytic Performance and Stability

**Figure 7:**
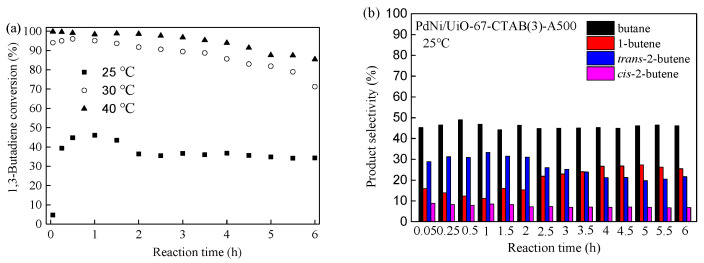

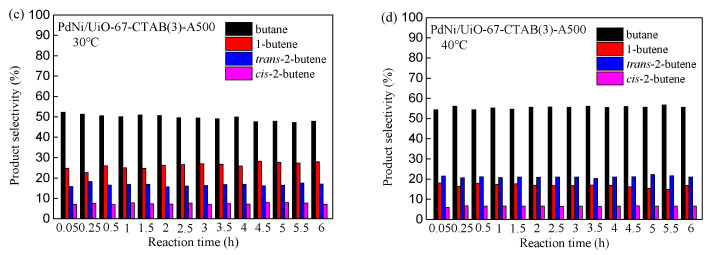
1,3-butadiene conversion profiles (**a**) and product selectivity histograms (**b**–**d**) over PdNi/UiO-67-CTAB(3)-A500 catalyst at different reaction temperatures (25–40 °C) (reaction conditions: 20 mL/min 1.0 vol% 1,3-butadiene balanced with N_2_, 10 mL/min 99.999% H_2_, catalyst quantity = 5 mg).

The catalytic performance of PdNi/UiO-67-CTAB(n)-A500 (*n* = 0, 3, 8, 13, 18) for the hydrogenation of 1,3-butadiene was investigated. The hydrogenation activity of the blank (without catalyst) can be neglected owing to the low 1,3-butadiene conversion (˂1%) at 25–40 °C. Figure 7 exhibits the 1,3-butadiene conversion and product selectivity of PdNi/UiO-67-CTAB(3)-A500 at different reaction temperatures (25–40 °C). At a low temperature of 25 °C, the conversion of 1,3-butadiene first increased, then decreased, and thereafter remained constant with the extension of the reaction time (Figure 7a). The initial conversion of 1,3-butadiene was only 4.7%; yet when the reaction time was 1 h, the conversion reached the maximum, up to 46.0%; the conversion was 34.2% within 6 h (Figure 7a). At a temperature of 30 °C, the conversion of 1,3-butadiene first increased and then decreased with values ranging from 71.3% to 96.1% (Figure 7a). At a relatively high reaction temperature of 40 °C, the conversion of 1,3-butadiene decreased with an increase in reaction time, and the conversions decreased from 99.9% to 85.4% (Figure 7a). The results displayed that the increase in the reaction temperature resulted in a continuous increase in 1,3-butadiene conversion. The ideal products of 1,3-butadiene hydrogenation were butenes (including 1-butene, *trans*-2-butene, and *cis*-2-butene), and butenes can be converted into butane by further hydrogenation [23,45]. The butane (44.2–49.0%) and total butene (51.0–55.8%) selectivities over PdNi/UiO-67-CTAB(3)-A500 changed slightly with the increase in reaction time at 25 °C (Figure 7b). The selectivity of *trans*-2-butene increased during the first 1 h of the reaction, then decreased from 1 h to 4 h, and then remained essentially constant from 4 h to 6 h (Figure 7b). However, the selectivity toward 1-butene displayed an opposite tendency compared to *trans*-2-butene over the entire reaction time range (Figure 7b). The selectivity of *cis*-2-butene remained approximately constant throughout the whole reaction time range, ranging from 6.8% to 8.9% (Figure 7b). When the reaction temperature rose to 30 °C, there was very little change in the distribution of products in the order of butane (47.3–52.3%) > 1-butene (22.7–27.9%) > *trans*-2-butene (15.7–18.3%) > *cis*-2-butene (7.0–8.0%) (Figure 7c). When the reaction temperature was further increased to 40 °C, the product distribution changed in the order of butane (54.3–56.7%) > *trans*-2-butene (20.3–22.3%) > 1-butene (15.0–18.1%) > *cis*-2-butene (6.1–6.7%) (Figure 7d). When the reaction temperature increased from 25 °C to 40 °C, the selectivity toward total butenes slightly decreased from 51.0–55.8% to 43.5–45.7%, and the selectivity toward butane increased from 44.2–49.0% to 54.3–56.5% (Figure 7). This may be due to the stronger adsorption of butenes that was generated on surface PdNi alloy nanoparticles at a higher temperature, resulting in deep hydrogenation [80,81]. Therefore, the higher reaction temperature promotes the secondary hydrogenation of butenes to butane [59]. The data of Figure 7 proved that the 1,3-butadiene conversion and selectivity of PdNi/UiO-67-CTAB(3)-A500 were related to the reaction temperature; as the reaction temperature increased, the 1,3-butadiene conversion of the catalyst increased, and the selectivity toward total butenes decreased.

**Figure 8:**
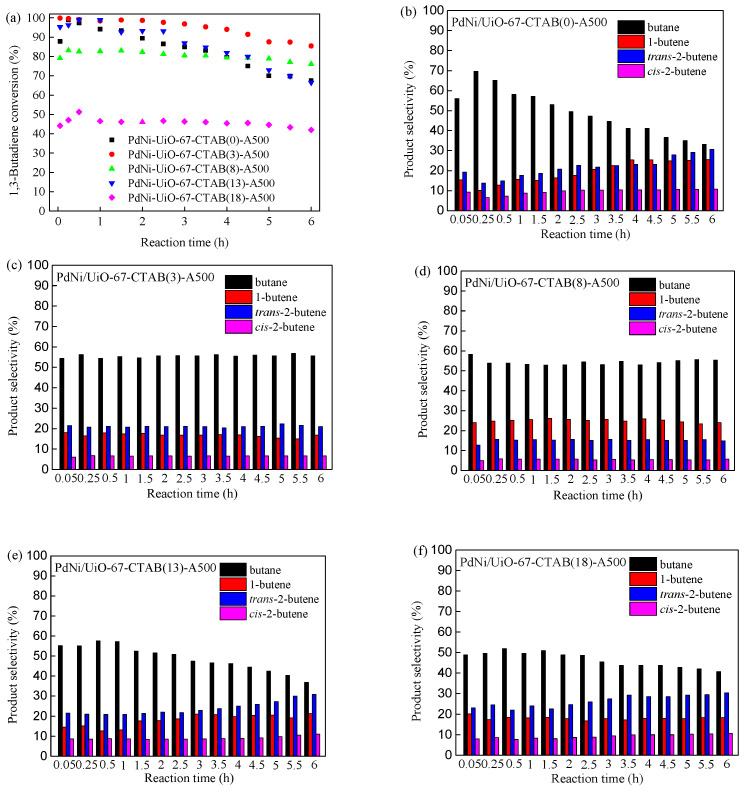
1,3-butadiene conversion profiles (**a**) and product selectivity histograms (**b**–**f**) over PdNi/UiO-67-CTAB(n)-A500 (*n* = 0, 3, 8, 13, 18) catalysts at 40 °C (reaction conditions: 20 mL/min 1.0 vol% 1,3-butadiene balanced with N_2_, 10 mL/min 99.999% H_2_, catalyst quantity = 5 mg).

The 1,3-butadiene conversions and product selectivities of PdNi/UiO-67-CTAB(n)-A500 (*n* = 0, 3, 8, 13, 18) catalysts for the selective hydrogenation of 1,3-butadiene are shown in Figure 8. Clearly, PdNi/UiO-67-CTAB(3)-A500 displayed the best performance for the selective hydrogenation of 1,3-butadiene. For the PdNi/UiO-67-CTAB(3)-A500 catalyst, the 1,3-butadiene conversion was maintained at about 99.0% at the first 2 h of the reaction, then gradually decreased as the reaction proceeded further, and the conversion of 1,3-butadiene dropped to 85.4% within 6 h (Figure 8a). The selectivity to total butenes changed slightly in the whole reaction process, and its value was between 43.5% and 45.7% (Figure 8c). For the PdNi/UiO-67-CTAB(0)-A500 catalyst, the conversion of 1,3-butadiene slightly increased during the first 0.25 h and then decreased significantly from 0.5 h to 6 h (Figure 8a). The selectivity of total butenes decreased during the first 0.25 h and then increased from 0.5 h to 6 h (Figure 8b). The conversion of 1,3-butadiene and the selectivity of total butenes were 67.6% and 66.9% after reaction 6 h at 40 °C for PdNi/UiO-67-CTAB(0)-A500, respectively (Figure 8a,b). The PdNi/UiO-67-CTAB(13)-A500 catalyst displayed a similar shaped conversion curve and catalytic performance to that of the PdNi/UiO-67-CTAB(0)-A500 catalyst (Figure 8a,e). The 1,3-butadiene conversion and selectivity to total butenes were 66.5% and 63.2% within 6 h at 40 °C for the PdNi/UiO-67-CTAB(13)-A500 catalyst, respectively (Figure 8a,e). For the PdNi/UiO-67-CTAB(8)-A500 catalyst, the 1,3-butadiene conversion slightly increased during the first 0.25 h of the reaction, leveling off from 0.5 h to 3.5 h, and slightly decreased from 4 h to 6 h (Figure 8a). The selectivity for total butenes changed slightly at all reaction times, with values ranging from 41.8% to 47.1% (Figure 8d). The PdNi/UiO-67-CTAB(18)-A500 catalyst displayed the lowest catalytic activity. 1,3-butadiene was slightly increased with reaction time during the first 0.5 h varying from 44.2% to 51.3% and slightly decreased from 1 h to 6 h (42.0–46.6%) (Figure 8a). The selectivity of total butenes ranged from 48.2% to 59.3% for the PdNi/UiO-67-CTAB(18)-A500 catalyst (Figure 8f). These results indicated that the crystal size, BET surface area, and pore volume of the UiO-67 template have little effect on the catalytic activity of the catalyst. However, the crystal morphology of the UiO-67 template has an important effect on the catalytic activity of the catalyst. The PdNi/UiO-67-CTAB(3)-A500 catalyst which was synthesized using UiO-67-CTAB(3) with uniform octahedral morphology as a template displayed the highest catalytic activity, while that of PdNi/UiO-67-CTAB(18)-A500 with 100% irregular polyhedral crystals was the worst.

A brief comparison of the catalytic performance of reported Pd-based catalysts for the hydrogenation of 1,3-butadiene is presented in Table 2. It can be seen that our prepared PdNi/UiO-67-CTAB(3)-A500 catalyst displayed excellent 1,3-butadiene hydrogenation performance as compared to the reported hydrogenation monometallic Pd catalysts (Table 2, entries 1–4) [9,82]. The addition of Ni to a Pd monometallic catalyst can change the structure and electronic properties of the Pd catalyst and improve 1,3-butadiene conversion and butene selectivity [18,19]. The PdNi/UiO-67-CTAB(3)-A500 catalyst displayed better hydrogenation activity than SiO_2_- and UiO-66-NH_2_-supported Pd-Ni bimetallic catalysts (Table 2, entries 1, 6, 11) [13,20]. However, our prepared catalyst PdNi/UiO-67-CTAB(3)-A500 displayed lower hydrogenation activity than Ni_1_-Pd_1_/ZnO, single-atom Au@Pd@SiO_2_, Pd–Cu/Mn_2_O_3_, and PdNi/UiO-66 (1:1) catalysts (Table 2, entries 1, 5, 8–10) [13,20,83,84]. Although our prepared PdNi/UiO-67-CTAB(3)-A500 catalyst exhibited higher 1,3-butadiene conversion than that of the Pd-Ag/ES catalyst for the hydrogenation of 1,3-butadiene, its selectivity for total butenes was lower (Table 2, entries 1, 7) [24].

**Figure 9:**
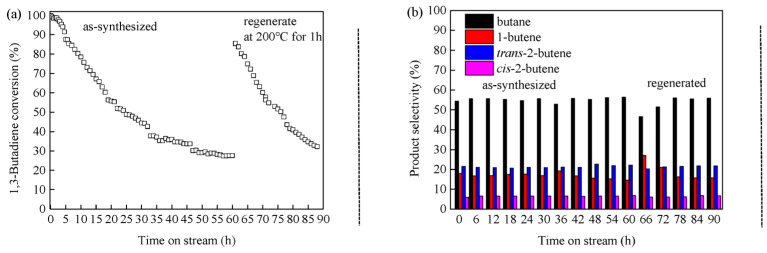
Stability and regenerate test of PdNi/UiO-67-CTAB(3)-A500 catalyst: (**a**) conversion; (**b**) product selectivity (reaction conditions: 20 mL/min 1.0 vol% 1,3-butadiene balanced with N_2_, 10 mL/min 99.999% H_2_, catalyst quantity = 5 mg, 40 °C).

**Figure 10:**
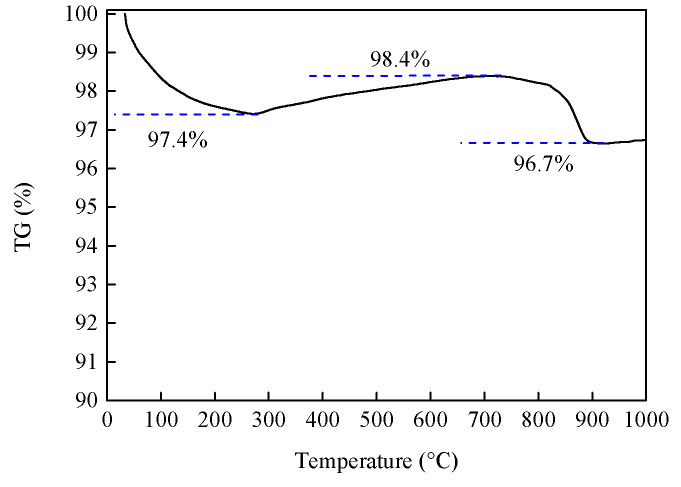
TG-DTA of PdNi/UiO-67-CTAB(3)-A500 after reaction at 40 °C for 24 h on stream.

The stability of catalysts is an important factor to be considered in industrial production [18,85]. Stability testing is highly necessary for the promotion and actual production of the catalyst. The durability of the PdNi/UiO-67-CTAB(3)-A500 catalyst was first examined for 60 h at 40 °C. Then, the catalyst was regenerated in N_2_ (12 mL/min) flowing at 200 °C for 1 h and tested for another 30 h. As shown in Figure 8a, PdNi/UiO-67-CTAB(3)-A500 was significantly deactivated during a total 60 h of the reaction time on the stream; the 1,3-butadiene conversion decreased from 99.9% to 27.6%. However, the product selectivity changed slightly at all reaction times (Figure 9b). The selectivity of total butenes varied between 43.6% and 45.7%. The regenerated PdNi/UiO-67-CTAB(3)-A500 catalyst displayed improved catalytic activity (Figure 9a). The conversion of 1,3-butadiene reached 85.5% after PdNi/UiO-67-CTAB(3)-A500 regenerated in flowing N_2_ at 200 °C for 1 h and then gradually decreased over the next 30 h. The total butene selectivity of the regenerated PdNi/UiO-67-CTAB(3)-A500 catalyst increased gradually at the first 18 h and then leveled off with the reaction. These results showed that the catalyst of PdNi/UiO-67-CTAB(3)-A500 can be regenerated at 200 °C. It was previously reported that carbon will be produced during the process of 1,3-butadiene hydrogenation, and the accumulation carbon will block the catalyst channels or deposit on the catalyst as films, thus leading to the deactivation of the catalyst [59,78,86,87]. The PdNi/UiO-67-CTAB(3)-A500 catalyst after the reaction at 40 °C for 24 h on the stream was characterized by TG in air atmosphere to analyze the deposited carbon. From the TG curve in Figure 10, the first weight loss stage from 30 °C to 275 °C owed to the release of physically adsorbed raw materials (1,3-butadiene and H_2_) and product materials (butane, 1-butene, *trans*-2-butene, and *cis*-2-butene). XPS displayed that Pd in PdNi/UiO-67-CTAB(3)-A500 existed as Pd^0^ and Pd^2+^ (Figure 5b). The TG curves showed a slight increase (1%) in weight from 275 °C to 700 °C, illustrating that Pd^0^ started to oxidize. Then, the TG curve had a decrease with the weight loss of 1.7% from 730 °C to 910 °C due to the deposited carbon being oxidized to generate CO_2_ and CO gases [88]. The calculated deposition carbon rate was 0.71 mg/(h·g_cat_).

## 3. Experimental Section

### 3.1. Synthesis of UiO-67-CTAB

UiO-67-CTAB(n) (*n* = 0, 3, 8, 13, 18) was prepared by using the surfactant CTAB assistant solvothermal method (CTAB: cetyltrimethylammonium bromide). First, ZrCl_4_ (0.64 mmol, 150.00 mg), 4,4-biphenyldicarboxylic acid (H_2_bpdc, 0.72 mmol, 175.00 mg), acetic acid (5 mL), and surfactant CTAB (0.135 g) were dissolved in 40 mL *N*,*N*′-dimethylformamide (DMF). Subsequently, the mixture was transferred into a 100 mL Teflon-lined autoclave reactor and maintained at 120 °C in a drying oven for 24 h. After natural cooling to room temperature, the white slurry was centrifuged. The obtained white solid was then washed three times with DMF and anhydrous methanol, respectively. Finally, the remaining white solid was dried under vacuum (vacuum degree: 0.1 MPa) at 50 °C for 5 h. The resultant powder was named as UiO-67-CTAB(3). The UiO-67-CTAB(8), UiO-67-CTAB(13), and UiO-67-CTAB(18) samples were synthesized by using a similar procedure, except using different CTAB concentrations (8, 13, and 18 g/L). The sample also synthesized by using a similar method without CTAB was named as UiO-67-CTAB(0) as the control.

### 3.2. Synthesis of PdNi/UiO-67-CTAB-A500

The PdNi/UiO-67-CTAB(n)-A500 (n: the concentration of CTAB; *n* = 0, 3, 8, 13, 18; A500: pyrolysis at 500 °C under an air atmosphere) catalyst was synthesized by the co-impregnation and pyrolysis method. Briefly, PdCl_2_ (0.047 mmol, 8.40 mg) and Ni(NO_3_)_2_·6H_2_O (0.085 mmol, 24.8 mg) were dissolved in 2 mL anhydrous ethanol. Then, the solution of PdCl_2_ and Ni(NO_3_)_2_·6H_2_O was dropwise added to 0.1 g UiO-67-CTAB(3). The suspension was left to age overnight at –4 °C and dried at 100 °C for 2 h in a vacuum oven with a vacuum degree of 0.1 MPa. The dry solid was placed in an alumina boat and pyrolyzed under an air atmosphere in a muffle furnace at 500 °C for 3 h (1 °C/min) to obtain PdNi/UiO-67-CTAB(3)-A500. The PdNi/UiO-67-CTAB(0)-A500, PdNi/UiO-67-CTAB(8)-A500, PdNi/UiO-67-CTAB(13)-A500, and PdNi/UiO-67-CTAB(18)-A500 catalysts were also synthesized in a similar procedure by the co-impregnation and pyrolysis method. The actual metal (Pd, Ni, and Zr) content of the catalysts as determined by ICP-OES is shown in Table S1.

### 3.3. Catalytic Activity Evaluation

The hydrogenation of 1,3-butadiene was carried out in a fixed bed using a quartz reactor with an internal diameter of 6 mm at 25–40 °C under atmospheric pressure. Typically, 5 mg of the catalyst was diluted with 500 mg of quartz sand (inert diluent, 40–80 mesh). The catalyst was pretreated in flowing N_2_ (8 mL/min) at the reaction temperature for 0.5 h. The reaction gas mixture was 1,3-butadiene/N_2_ (1.0 vol.%, 20 mL/min) and H_2_ (10 mL/min). The space velocity reached 360,000 mL/(h·g_cat_) which could eliminate mass-transfer limitation during the 1,3-butadiene hydrogenation. The gas stream at the reactor outlet was analyzed using online GC-6890 (Purkinje General Instrument Co., Ltd., Beijing, China) equipped with an Al_2_O_3_ capillary column (30 m×0.53 mm×10 mm). Carbon balance was 97% ± 3%. 1,3-butadiene conversion (*X*_1,3-*BD*_) and selectivities (*S*_i_) of butane, 1-butene, *trans*-2-butene, and *cis*-2-butene were calculated using the following equations:

$$X_{1,3-BD}(\%)=\frac{{\left[1,3-BD\right]}_{i}-{\left[1,3-BD\right]}_{f}}{{\left[1,3-BD\right]}_{i}}\times 100$$

$$S_{i}(\%)=\frac{C_{i}}{\sum_{i}C_{i}}\times 100$$

where [1,3-*BD*]*_i_* is the initial molar flow rate of 1,3-*BD* (mmol/min), [1,3-*BD*]*_f_* is the final molar flow rate of 1,3-*BD* (mmol/min), C_i_ is the product (such as butane) molar flow rate, and $\sum_{i}C_{i}$ is the total product (containing butane, 1-butene, *trans*-2-butene, and *cis*-2-butene) molar flow rate.

## 4. Conclusions

We have studied the selective hydrogenation of 1,3-butadiene on PdNi/UiO-67-CTAB(n)-A500 (*n* = 0, 3, 8, 13, 18) catalysts. Catalytic activity strongly depends on the crystal morphology of the UiO-67 template for 1,3-butadiene hydrogenation. The highest catalytic activity was observed over the PdNi/UiO-67-CTAB(3)-A500 catalyst which was synthesized using UiO-67 with a uniform octahedral morphology as the template for 1,3-butadiene selective hydrogenation. Catalytic activity increased with increasing reaction temperature for PdNi/UiO-67-CTAB(3)-A500, whereas the selectivity to total butenes slightly decreased. The higher reaction temperature could promote the secondary hydrogenation of butenes to butane. The 1,3-butadiene conversion and the selectivity of total butenes were 98.4% and 44.8% at 40 °C within 1 h for the PdNi/UiO-67-CTAB(3)-A500 catalyst, respectively. The catalyst of PdNi/UiO-67-CTAB(3)-A500 can be regenerated in flowing N_2_ at 200 °C. Carbon deposited on the catalyst during the 1,3-butadiene hydrogenation process was the main reason for its deactivation. This work is valuable for the high-efficiency bimetallic catalyst’s development on the selective hydrogenation of 1,3-butadiene.

## Figures and Tables

**Table 1:** Comparison of physicochemical properties of different samples.

Entry	Sample	S_BET_ (m^2^/g)	V_total_ (cm^3^/g)
1	UiO-67-CTAB(0)	686.1	0.40
2	UiO-67-CTAB(3)	264.9	0.17
3	UiO-67-CTAB(8)	1402.2	0.73
4	UiO-67-CTAB(13)	1611.8	0.83
5	UiO-67-CTAB(18)	1309.1	0.70
6	PdNi/UiO-67-CTAB(0)-A500	60.9	0.09
7	PdNi/UiO-67-CTAB(3)-A500	54.6	0.09
8	PdNi/UiO-67-CTAB(8)-A500	66.7	0.11
9	PdNi/UiO-67-CTAB(13)-A500	62.4	0.09
10	PdNi/UiO-67-CTAB(18)-A500	54.3	0.09

**Table 2:** The catalytic performance of reported Pd-based catalysts in the hydrogenation of 1,3-butadiene.

**Entry**	**Catalyst**	**Pd Loading (wt%)**	**Pd Size (nm)**	**T (°C)**	**Conv. (%)**	**Sel. (%)**	**Ref.**
1	PdNi/UiO-67-CTAB(3)-A500	6.5	6.7	40	98.4	44.8	this work
2	Pd/pollen-C-500	1	112.6	50	23.0	97.9	9
3	1PdGONE	1	8.9	50	15	88	82
4	2PdG	2	4.9	50	89	27	82
5	Ni_1_-Pd_1_/ZnO	-	3.6	35	98.8	90.0	13
6	Ni_1_-Pd_1_/SiO_2_	-	3.2	35	44.0	93.8	13
7	Pd-Ag/ES	0.5	3.7	45	95.0	95.8	24
8	Au@Pd@SiO_2_	0.02	monodisperse	35	98.0	80.0	83
9	Pd–Cu/Mn_2_O_3_	1	3.8	50	99.1	92.0	84
10	PdNi/UiO-66 (1:1)	2.65	7.1	40	99.8	84.5	20
11	PdNi/UiO-66-NH_2_ (1:1)	2.51	4.6	40	15.6	99.5	20

## Data Availability

The original contributions presented in the study are included in the article and Supplementary Materials, further inquiries can be directed to the corresponding authors.

## Supplementary Materials

The following supporting information can be downloaded at https://www.mdpi.com/article/10.3390/molecules29102217/s1, Materials and Methods: materials, characterization and equipment; Table S1: The actual metal content of as-synthesized catalysts analyzed by ICP-OES; Figure S1: TEM photographs and PdNi nanoparticle distributions of PdNi/UiO-67-CTAB(0)-A500 (a,b), PdNi/UiO-67-CTAB(3)-A500 (c,d), PdNi/UiO-67-CTAB(8)-A500 (e,f), PdNi/UiO-67-CTAB(13)-A500 (g,h), and PdNi/UiO-67-CTAB(18)-A500 (i,j).
