# Supplementary material for: MOF-Derived ZrO2-Supported Bimetallic Pd–Ni Catalyst for Selective Hydrogenation of 1,3-Butadiene
Source: Molecules. 2024 May 9;29(10):2217. doi: 10.3390/molecules29102217 (PMC11123826; doi:10.3390/molecules29102217)
Supplement: Supplementary file 1 [file molecules-29-02217-s001.zip › molecules-2947297-supplementary.pdf]

MOF-Derived ZrO_2-Supported Bimetallic Pd–Ni Catalyst for Selective Hydrogenation of 1,3-Butadiene

Ying Liu, Lili Liu*, Leyuan Wang, Miaoliang Zang, Lei Li and Yunkai Zhang

School of Chemistry & Chemical Engineering and Environmental Engineering, Weifang University, Weifang 261061, P.R. China; liuying@wfu.edu.cn (Y.L.); 15264543037@163.com (L.W.); 13356710254@163.com (M.L.); 15863610289@163.com(L.L.); 17753624747@163.com (Y.Z.)

*Correspondence: liulili122@126.com (L.L.)

1. Materials and Methods

1.1 Materials

$PdCl_2$ (Pd≥59.5%), $ZrCl_4$ (98%), 4,4'-biphenyl dicarboxylic acid (H_2bpdc, 97%), and acetic acid (99.5%) were obtained from Innochem Science & Technology Co. Ltd. (Beijing, China). $Ni(NO_3)_2·6H_2O$ (99.99%), N,N'-dimethylformamide (DMF, 99.5%), cetyltrimethylammonium bromide (CTAB, 99.0%), methanol (99.5%), and ethanol (99.7%) were purchased from Aladdin Reagent Co. Ltd. (Shanghai, China). 1,3-butadiene/N_2 (1.0vol%) and H_2 (99.99%) were supplied by Hengan Gas factory (Anqiu, China).

1.2 Characterization and Equipment

The samples were characterized by powder X-ray diffraction (PXRD, Brüker D8, Germany) using Cu Kα radiation (λ = 1.54060 Å), X-ray photoelectron spectroscopy (XPS, AXIS ULTRADLD, Shimadzu, Japan), and inductively coupled plasma optical emission spectrometry (ICP-OES, Optima 5300DV, Perkin-Elmer, USA). The morphologies of the UiO-67-CTAB(n) (n=0, 3, 8, 13, 18) were monitored by scanning electron microscopy (SEM, Quanta 450 FEI, Graz, Austria) and transmission electron microscopy (TEM, Jeol JEM-1210, Jeol, Tokyo, Japan). Energy dispersive X-ray spectroscopy (EDS) was performed on the X-MaxN 80T IE250 instrument (Oxford, UK). Textural properties of the samples were investigated through N_2 sorption at 77 K and were performed utilizing an automatic physical adsorption instrument (ASAP2460, Atlanta, Georgia, USA). The samples were treated at 150°C for 18 h under vacuum (0.1 MPa) before measurements. The specific areas and pore size distributions were determined using Brunauer-Emmett-Teller (BET) method and density functional theory (DFT) method, respectively. Raman spectra of PdNi/UiO−67−CTAB(3)−A500 catalyst after reaction at 40°C for 60 h on stream were obtained using a Horiba LabRAM HR Evolution Raman microscope (Japan). Raman spectra were abtained using a laser power of 2 mW at λ_{exc}=532 nm with a collection time of 40 s by averaging 20 scans. The thermogravimetric analysis measurements were performed on a Rigaku TG-DTA 8122 simultaneous thermal analyzer (Japan) with a heating rate of 10°C/min from room temperature to 1000°C in Air atmosphere.

Table S1. The actual metal content of as-synthesized catalysts analyzed by ICP-OES.

entry	catalyst	Pd content (wt%)	Ni content (wt%)	Zr content (wt%)
1	PdNi/UiO−67−CTAB(0)−A500	6.2	5.7	49.8
2	PdNi/UiO−67−CTAB(3)−A500	6.5	5.4	49.4
3	PdNi/UiO−67−CTAB(8)−A500	6.4	5.5	49.4
4	PdNi/UiO−67−CTAB(13)−A500	6.5	5.4	49.2
5	PdNi/UiO−67−CTAB(18)−A500	6.7	5.5	49.9

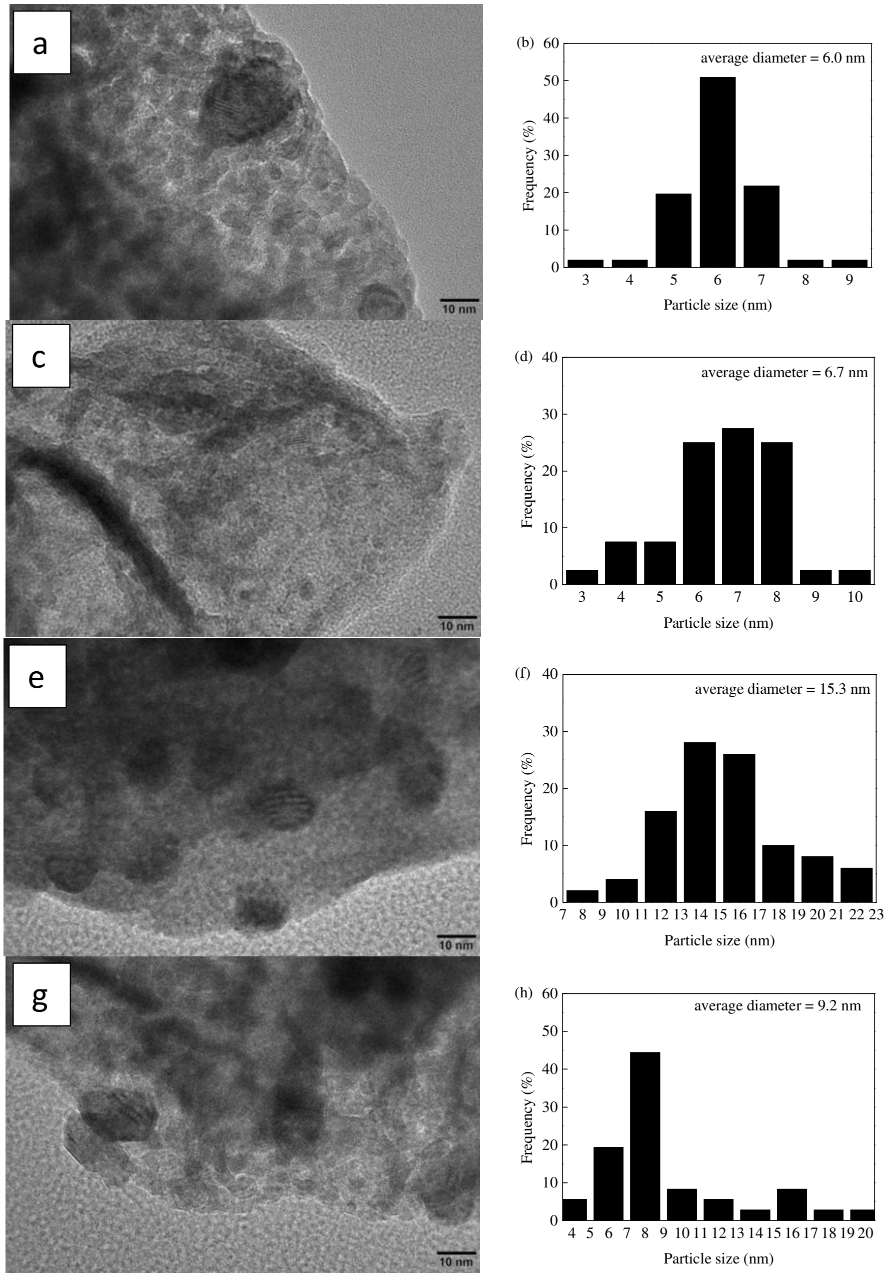
a
10 nm
(b)
average diameter = 6.0 nm
Frequency (%)
Particle size (nm)
c
10 nm
(d)
average diameter = 6.7 nm
Frequency (%)
Particle size (nm)
e
10 nm
(f)
average diameter = 15.3 nm
Frequency (%)
Particle size (nm)
g
10 nm
(h)
average diameter = 9.2 nm
Frequency (%)
Particle size (nm)

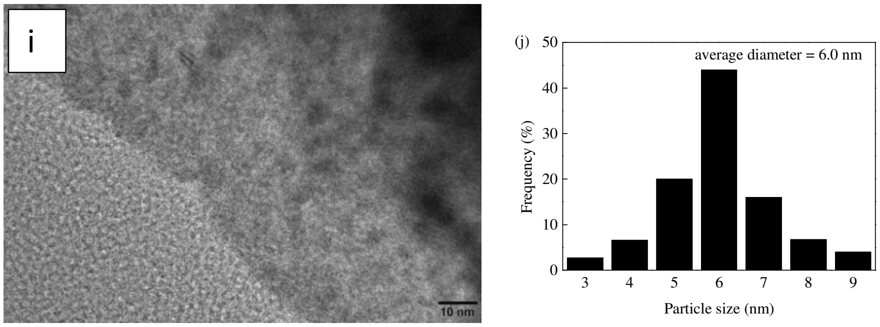

Figure S1. TEM photographs and PdNi nanoparticle distributions of PdNi/UiO−67−CTAB(0)−A500 (a, b), PdNi/UiO−67−CTAB(3)−A500 (c, d), PdNi/UiO−67−CTAB(8)−A500 (e, f), PdNi/UiO−67−CTAB(13)−A500 (g, h), PdNi/UiO−67−CTAB(18)−A500 (i, j)
